# Epigenome-wide association study of seizures in childhood and adolescence

**DOI:** 10.1186/s13148-019-0793-z

**Published:** 2020-01-08

**Authors:** Doretta Caramaschi, Charlie Hatcher, Rosa H. Mulder, Janine F. Felix, Charlotte A. M. Cecil, Caroline L. Relton, Esther Walton

**Affiliations:** 1 Bristol Medical School, Population Health Sciences, University of Bristol, 5 Tyndall Avenue, Bristol, BS8 1UD UK; 2 Medical Research Council Integrative Epidemiology Unit, University of Bristol, Oakfield House, Oakfield Grove, Bristol, BS8 2BN UK; 3 Institute of Education and Child Studies, Leiden University, Pieter de la Court building, Wassenaarseweg 52, 2333 AK Leiden, The Netherlands; 4 Generation R Study Group, Erasmus MC, University Medical Center Rotterdam, Doctor Molewaterplein 40, 3015 GD Rotterdam, The Netherlands; 5 Department of Child and Adolescent Psychiatry/Psychology, Erasmus MC, University Medical Center Rotterdam, Doctor Molewaterplein 40, 3015 GD Rotterdam, The Netherlands; 6 Department of Pediatrics, Erasmus MC, University Medical Center Rotterdam, Doctor Molewaterplein 40, 3015, GD Rotterdam, the Netherlands; 7 Department of Epidemiology, Erasmus MC, University Medical Center Rotterdam, Doctor Molewaterplein 40, 3015 GD Rotterdam, The Netherlands; 8 Department of Psychology, University of Bath, Bath, BA2 7AY UK

**Keywords:** Seizures, Epilepsy, Epigenetics, DNA methylation, ALSPAC, Illumina 450 K, Mendelian randomization, Generation R Study

## Abstract

The occurrence of seizures in childhood is often associated with neurodevelopmental impairments and school underachievement. Common genetic variants associated with epilepsy have been identified and epigenetic mechanisms have also been suggested to play a role. In this study, we analyzed the association of genome-wide blood DNA methylation with the occurrence of seizures in ~ 800 children from the Avon Longitudinal Study of Parents and Children, UK, at birth (cord blood), during childhood, and adolescence (peripheral blood). We also analyzed the association between the lifetime occurrence of any seizures before age 13 with blood DNA methylation levels. We sought replication of the findings in the Generation R Study and explored causality using Mendelian randomization, i.e., using genetic variants as proxies. The results showed five CpG sites which were associated cross-sectionally with seizures either in childhood or adolescence (1–5% absolute methylation difference at p_FDR_ < 0.05), although the evidence of replication in an independent study was weak. One of these sites was located in the *BDNF* gene, which is highly expressed in the brain, and showed high correspondence with brain methylation levels. The Mendelian randomization analyses suggested that seizures might be causal for changes in methylation rather than vice-versa. In conclusion, we show a suggestive link between seizures and blood DNA methylation while at the same time exploring the limitations of conducting such study.

## Background

Seizures are episodes of abnormal excessive or synchronous neuronal activity in the brain. When associated with a febrile illness, they affect 2–4% of children under 6 years of age in Europe and the USA, with the highest incidence in underdeveloped and rural areas reaching 14% in some areas [1, 2]. The most common age for seizures to occur is at 18 months of age and children that experienced seizures are at risk of developing epilepsy. The incidence of epilepsy in children ranges from 41 to 187/100,000 persons per year [2]. Seizures and epilepsy are associated with neurodevelopmental conditions, such as autism spectrum disorders [3], attention-deficit hyperactivity disorder, and cognitive impairment [4–7]. Moreover, epilepsy with or without intellectual impairment is associated with low academic achievement [8].

The two largest genome-wide association studies to date (~ 8600 individuals with epilepsy versus ~ 26000 controls and ~ 15,200 individuals with epilepsy versus ~ 29,600 controls) have identified a total of 24 genetic variants associated with epilepsy [9, 10]. Some of these loci are located in the proximity of candidate genes for epilepsy, for instance those coding for ion-channel subunits, and their relevance for epilepsy is supported by other research in humans and animals. As it is likely that other factors may also underlie the disease, it has been suggested that epigenetic mechanisms such as DNA methylation are also involved in the onset of seizures [11]. In line with this hypothesis, genetic markers for epilepsy were found to be enriched in histone modification markers, suggesting epigenetic regulation of gene transcription [9]. The association between seizures and DNA methylation has been investigated in studies involving humans and other animals, although these studies relied on small sample sizes or on a candidate gene approach. There are different types of epilepsy syndromes depending on the age of onset (e.g., childhood or adolescence), whether the seizures are predominantly characterized by a focal or generalized onset, and whether there are known causes (e.g., genetic or trauma) [12]. Mesial temporal lobe epilepsy is one of the most common and most studied forms of epilepsy [13]. A recent study comparing blood DNA methylation in 30 adult patients with mesial temporal lobe epilepsy and 30 controls identified 216 differentially methylated sites between the two groups, including sites on genes involved in ion binding and metabolic activity [14]. DNA methylation differences have also been observed in lymphoblastoid cell lines derived from epileptic patients, both when DNA methylation was measured globally using antibody capture and in the *BRD2* gene promoter [15]. Another study that reanalyzed these data discovered differential DNA methylation in non-coding RNAs [16]. Furthermore, alterations in DNA methylation were present in the hippocampus of epileptic patients compared to controls [17]. A study adopting a rat model of chronic epilepsy corroborated these findings by revealing genome-wide differences in DNA methylation compared to control rats [18].

Typically, in association studies, it is difficult to assess the causality of any identified association due to the potential for confounding and/or for reverse causation. Socioeconomic status, for instance, is associated with genome-wide changes in DNA methylation [19] and with increased risk for seizures/epilepsy [20], suggesting that socioeconomic factors could be confounding the association between DNA methylation and seizures. With respect to reverse causation, case-control studies that examined DNA methylation after epilepsy had already been diagnosed might have observed changes that were directly caused by the seizure events. For instance, laboratory animal studies have shown altered gene expression after induced seizures [21]. Mendelian randomization, a technique that uses the genetic information associated with an exposure to estimate the causal effect of the exposure on an outcome, can circumvent these limitations under certain assumptions [22].

**Fig. 1:**
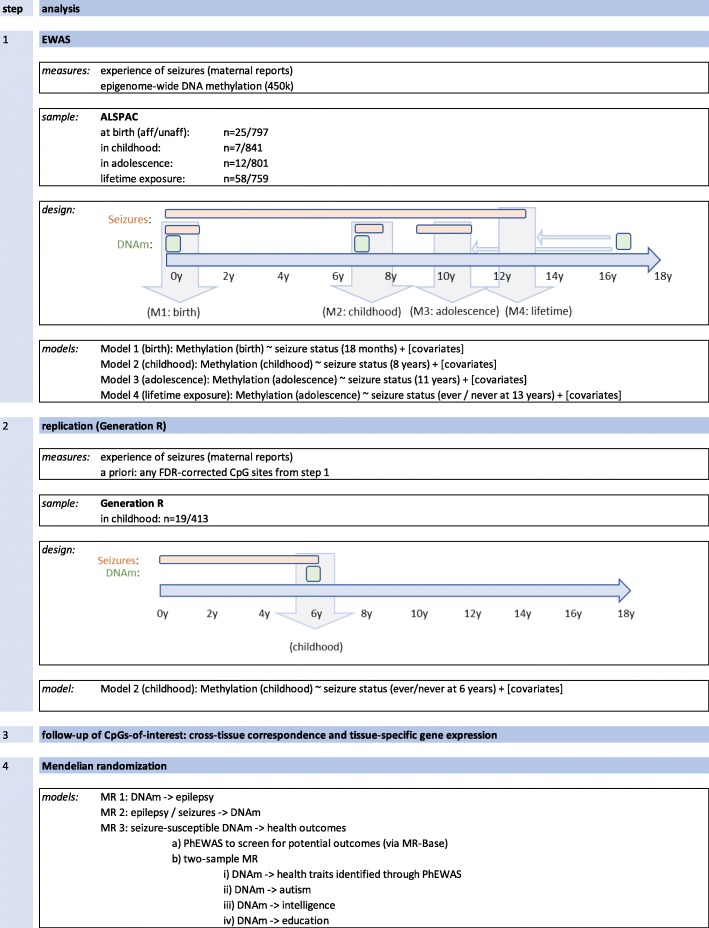
Analysis overview

In this study, we (1) investigated the genome-wide association of DNA methylation with the occurrence of seizures from birth throughout childhood and adolescence in peripheral blood samples from a prospective birth cohort, (2) carried out replication analyses in an independent study sample, (3) explored the correspondence with brain tissue DNA methylation at the same genomic locations to investigate whether these associations have neurodevelopmental relevance, and (4) performed bi-directional Mendelian randomization to unravel causal links between peripheral blood DNA methylation and seizures. Finally, we explored the potential health consequences of a seizure-associated DNA methylation profile. For an overview on our analysis plan, see Fig. 1.

## Materials and methods

### Study population

The discovery analyses were conducted in the Avon Longitudinal Study of Parents and Children (ALSPAC), a large prospective cohort study that recruited 14,541 pregnant women, resident in Avon, UK with expected delivery dates between the 1st of April 1991 and the 31st of December 1992 [23, 24]. Of these initial pregnancies, there were 14,062 live births and 13,988 children who were alive at 1 year of age. The study website contains details of all the data that are available through a fully searchable data dictionary (http://www.bris.ac.uk/alspac/researchers/data-access/data-dictionary/).

Written informed consent has been obtained for all ALSPAC participants. Ethical approval for the study was obtained from the ALSPAC Ethics and Law Committee and the Local Research Ethics Committees.

### Seizure data

The diagnosis of seizures and epilepsy is usually based on the pattern of seizures, age of onset, and electroencephalographic and imaging features, and potentially the history of comorbidities as well as genetic and/or metabolic screening [25]. In our ALSPAC population study, we relied on data taken from questionnaires administered to the mothers at four time points: 18 months, 8 years, 11 years, and 13 years. The following questions were asked at each of these ages:

18 months: “Has he/she ever had any form of convulsion/fit/seizure or other turn in which consciousness was lost or any other part of the body made an abnormal movement?’,

8 years: “Has child had a convulsion/fit/seizure since 7th birthday?”,

11 years: “Has child had a convulsion/fit/seizure where consciousness or abnormal movement was lost since 9th birthday?”, and

13 years: “Has she ever had a seizure, fit or a convulsion?”.

This allowed us to address proximal and distal (i.e., lifetime until age 13) associations between DNA methylation and experience of seizures. The seizures reported in our study could have included any type of seizure that might not be considered epilepsy, such as febrile seizures typically experienced before 6 years of age and single occurrences provoked by trauma. Alternatively, they could be recurring seizures of the types typical of an epilepsy syndrome. However, we did not have access to the information on the clinical diagnoses in relation to epilepsy in the study participants.

### DNA methylation data

In ALSPAC, blood from 1018 mother-child pairs were selected for analysis as part of the Accessible Resource for Integrative Epigenomic Studies (ARIES, http://www.ariesepigenomics.org.uk/) [26]. Following DNA extraction, samples were bisulphite converted using the Zymo EZ DNA Methylation™ kit (Zymo, Irvine, CA, USA), and genome-wide methylation was measured using the Illumina Infinium HumanMethylation450 (HM450) BeadChip. The arrays were scanned using an Illumina iScan, with initial quality review using GenomeStudio. ARIES was pre-processed and normalized using the *meffil* R package [27]. ARIES consists of from mother-child pairs measured at five time points (three time points for children: birth, childhood, and adolescence; and two for mothers: during pregnancy and at middle age), although only children’s profiles were used in the current study. Low quality profiles were removed from further processing, and the remaining 4593 profiles were normalized using the Functional Normalization algorithm [28] with the top 10 control probe principal components. Full details of the pre-processing and normalization of ARIES have been described previously [27]. Further pre-processing specific to the current study included removal of probes not passing background detection (*p* > 0.05) and probes on the X or Y chromosome. To reduce the impact of outliers, we set methylation data points outside the 3× inter-quartile-range from the 25th and the 75th percentiles to missing. The total number of probes available for analyses were *N* = 468,828 at birth; *N* = 471,092 at childhood; and *N* = 470,480 at adolescence.

### Epigenome-wide association analyses (EWAS)

In ALSPAC, the final sample size at birth was *N* = 822 (25 cases and 797 controls); at childhood *N* = 848 (7 cases and 841 controls) and *N* = 813 (12 cases and 801 controls) at adolescence. The final sample only contained singletons and no siblings. Only 2–3 cases overlapped across time points (Additional file 1: Figure S1). This is to be expected as certain types of seizures, such as febrile ones, are more common in the first years of life and children diagnosed with epilepsy might have received treatment to prevent further seizures. Moreover, the age of onset of some forms of epilepsy is between childhood and adolescence [29], explaining why some children might have had their first seizures in their adolescence. Fifty-eight out of a total of *N* = 817 adolescents reported a lifetime experience of seizures.

We carried out four analyses. In analysis 1–3, we modelled methylation at birth (model 1), childhood (model 2), and adolescence (model 3) as the outcome and seizure status (measured closest to each methylation time point) as the exposure. To investigate the temporal sensitivity of associations, we ran a final analysis (model 4), in which we modelled lifetime seizure status (ever/never) at 13 years as the exposure and methylation at adolescence as the outcome (model 4). In all models (including birth), methylation was defined as the outcome regardless of temporal order to keep model estimates consistent and comparable. These EWAS were carried out in R version 3.3.1 using the CpGassoc package [30].

All models were adjusted for age (when DNA methylation samples were taken), sex, prenatal maternal smoking (Yes/No), and maternal education (university degree Yes/No), each derived from ALSPAC maternal and childhood questionnaires. The model using cord blood data was additionally adjusted for gestational age and birthweight. Unknown confounders and batch were adjusted using surrogate variable (SV) analysis [31]. Additionally, we adjusted for cell counts using the Houseman method for the childhood and adolescence timepoints [32] and the Andrews and Bakulski method for cord blood [33].

To summarize, the following models were applied:

Model 1: Methylation (cord) ~ seizure status (18 months) + age + sex + birthweight + gestational age + maternal prenatal smoking + maternal education + nucleated red blood cells + granulocytes + monocytes + natural killer cells + B cells + CD4(+)T cells + CD8(+)T cells +SV1 + … + SV15

Model 2: Methylation (childhood) ~ seizure status (8 years) + age + sex + maternal prenatal smoking + maternal education + granulocytes + monocytes + natural killer cells + B cells + CD4(+)T cells + CD8(+)T cells + SV1 + … + SV13

Model 3: Methylation (adolescence) ~ seizure status (11 years) + age + sex + maternal prenatal smoking + maternal education + granulocytes + monocytes + natural killer cells + B cells + CD4(+)T cells + CD8(+)T cells + SV1 + … + SV14

Model 4: Methylation (adolescence) ~ seizure status (ever/never at 13 years) + age + sex + maternal prenatal smoking + maternal education + granulocytes + monocytes + natural killer cells + B cells + CD4(+)T cells + CD8(+)T cells + SV1 + … + SV14

To correctly multiple testing, we present both Bonferroni (0.05/number of probes) and FDR-corrected results.

### Replication analyses

All CpG sites that were associated with seizures below at least an FDR-correction threshold in ALSPAC were analyzed in an independent cohort to assess replication. The Generation R Study is a population-based prospective cohort study conducted in Rotterdam, the Netherlands, that recruited 9778 pregnant women with an expected delivery date between April 2002 and January 2006. A total of 9749 children were born from these pregnancies, and extensive data and biological samples are available from the children and their mothers [34]. DNA methylation was measured in peripheral blood of 469 children aged 6 years (all singletons), using the Infinium HumanMethylation450 (HM450) BeadChip as in ALSPAC. Preparation and normalization of the BeadChip array was performed according to the CPACOR workflow in R [35] and methylation data points lower than the 25th percentile − 3 × IQR and higher than the 75th percentile + 3 × IQR were excluded. In Generation R, seizure events were measured using the answer “yes” to the question “During the past 5/6 years, did your child ever have a seizure/febrile convulsion?” asked to the mothers when the children were 6 years of age. The final sample size was *N* = 432, with 19 participants affected by seizures and 413 unaffected (Additional file 1: Table S1). Linear models similar to model 2 were run in Generation R on EWAS FDR-corrected methylation sites and on *BDNF* probes. The covariates were measured and categorized similarly to the analyses carried out in ALSPAC. The results in ALSPAC and Generation R where also meta-analyzed using METAL [36], using inverse variance weighting.

### Mendelian randomization analyses

To assess the causal link between DNA methylation and the occurrence of seizures, we performed two-sample Mendelian randomization (MR) using the EWAS results for the CpG sites with FDR-corrected *p* values < 0.05 in ALSPAC. Two-sample MR was performed using the MR-Base online platform (http://www.mrbase.org/, last accessed 06-06-2018) [37], the MRInstruments R package (https://github.com/MRCIEU/MRInstruments, last accessed 06-06-2018), and the TwoSampleMR R package (https://github.com/MRCIEU/TwoSampleMR, last accessed 06-06-2018).

Three MR analyses were performed. (1) To investigate the causal effect of DNA methylation on the risk of epilepsy, we performed two-sample MR with DNA methylation as exposure and a diagnosis of epilepsy as outcome. For the genotype-exposure associations, we searched for methylation quantitative trait loci (mQTLs), i.e., genetic variants that are associated in *cis* with DNA methylation (i.e., within 1 Mb either side from the CpG site), using the mQTL database (http://www.mqtldb.org/, last accessed 06 June 2018) [38], restricting the search to the time point, at which the CpG site was associated with seizure status. For the genotype-epilepsy association, we used the summary statistics for ICD-9 and ICD-10 codes for epilepsy or seizure in MR-Base generated on UKBiobank data (last accessed 04 December 2018). (2) To analyze the causal effect of risk for seizure/epilepsy on DNA methylation (i.e., reverse causation), we performed two-sample MR with epilepsy diagnosis or febrile/vaccine-related seizures as exposure and DNA methylation as outcome. For the genotype-exposure associations, we used the summary statistics for the genome-wide significant SNPs from a published GWAS meta-analysis on all epilepsies, focal epilepsy, and genetic generalized epilepsy [10] and from a published GWAS on febrile and MMR-vaccine-related seizures (focussing on 6 replicated genome-wide significant SNPs in Table1 of the original article) [39]. Odds ratios (OR) and confidence intervals were reverted to log-odds and standard errors to use in the two-sample MR analysis. The summary statistics for the genotype-outcome associations were drawn from the mQTL database (http://www.mqtldb.org/, last accessed 06 June 2018) [38]. (3) To analyze the causal effect of seizure-susceptible DNA methylation (exposure) on other health outcomes, we first performed a hypothesis-free PheWAS using the mQTLs for CpG sites associated with seizures and the MR-Base online PheWAS tool to screen for potentially affected health outcomes. Then, we performed two-sample MR with methylation as exposure and the health outcomes identified by the PheWAS to estimate the magnitude of the effect. We also performed a hypothesis-driven two-sample MR on other neurodevelopmental outcomes previously found to associate with seizures, i.e., autism, intelligence, and education.

## Results

For a flowchart and overview of all results, see Additional file 1:Figure S2.

### Sample description

At 18 months, there were *n* = 25 children with the experience of seizures since birth and *n* = 797 without (Table 1). Groups were comparable with respect to sex, birthweight, gestational age, as well as maternal education and smoking behavior during pregnancy. During childhood, *n* = 7 children had experienced seizures between 7 and 8 years of age while 841 had not. Age at blood draw was slightly higher in the children with seizures (Table 1). Approaching adolescence, seizures had been reported in *n* = 12 children, while *n* = 801 children had not experienced seizures between 9 and 11 years of age. There were slightly more females in the seizure group. Fifty-eight adolescents reported a lifetime experience of seizures, while *n* = 759 had never experienced seizures. Groups were comparable with respect to maternal smoking behavior, maternal education, and age at blood draw. For correlation plots on seizure rates and all covariates included in the final models specific to each time point, see Additional file 1: Figures S3

**Table 1:** ARIES sample characteristics

Characteristic	Birth			Childhood			Adolescence			Adolescence (lifetime exposure)		
	With seizures	Without seizures	Total	With seizures	Without seizures	Total	With seizures	Without seizures	Total	With seizures (ever)	Without seizures (ever)	Total
*N*	25	797	822	7	841	848	12	801	813	58	759	817
Sex (%F)	44%	51%	51%	ND	50%	51%	ND	51%	52%	52%	51%	51%
Age at methylation in years (SD)	NA			7.69 (0.36)	7.44 (0.13)	7.45 (0.14)	17.65 (0.86)	17.11 (1.04)	17.12 (1.04)	17.16 (1.01)	17.10 (1.05)	17.10 (1.04)
Age at seizure assessment in years (SD)	1.54 (0.12)	1.52 (0.06)	1.52 (0.07)	8.60 (0.03)	8.66 (0.16)	8.66 (0.16)	11.70 (0.08)	11.71 (0.12)	11.71 (0.12)	13.12 (0.09)	13.15 (0.17)	13.43 (1.05)
Birthweight in g (SD)	3498.80 (545.54)	3495.08 (478.18)	3495.20 (480.00)	NA			NA			NA		
Gestational age in weeks (SD)	39.80 (1.50)	39.55 (1.48)	39.56 (1.48)									
Maternal smoking (%Y)	ND	13%	14%	ND	12%	13%	ND	13%	13%	14%	13%	13%
Maternal education (%Uni)	32%	20%	21%	ND	21%	21%	ND	22%	22%	28%	22%	22%

*ND* not disclosed due to cohort restrictions, *NA* not applicable

### Epigenome-wide association analyses

We did not identify any CpG sites that fell below a Bonferroni or FDR-adjusted *P* value threshold at birth (Table 2 and Additional file 1: Figure S4). In childhood, two CpG sites were associated with seizure status at an FDR-corrected *p* value < 0.05 (cg10541930: beta = − 0.010, SE = 0.002, uncorrected *p* value = 4.32 × 10^−8^ and FDR = 0.020; cg25557432: beta = 0.014, SE = 0.003, uncorrected *p* value = 1.82 × 10^−7^ and FDR = 0.043), of which cg10541930 was also below the Bonferroni threshold (0.05/470,489 = 1.06 × 10^−7^). The first CpG is located in an intergenic region, at the transcription start site for a non-coding RNA, while the second lies upstream of the gene *MACROD2* involved in DNA repair.

**Table 2:** EWAS results. Top CpG sites for the three time-points: birth, childhood, and adolescence (cross-sectional and lifetime exposure)

Time point	ProbeID	Beta	SE	*P* value	FDR	Bonferroni	Chromosome	Position	Gene
Birth	cg07504545	− 0.042	0.008	2.32 × 10^−07^	0.109	0.109	1	203456019	*PRELP*
	cg04977770	− 0.054	0.011	7.15 × 10^−07^	0.168	0.335	17	79846763	*THOC4*
	cg23625106	− 0.023	0.005	1.47 × 10^−06^	0.193	0.689	8	61789727	
	cg08461451	− 0.036	0.008	1.73 × 10^−06^	0.193	0.812	19	2295092	*LINGO3*
	cg06367149	0.057	0.012	2.08 × 10^−06^	0.193	0.973	15	61254575	*RORA*
	cg13396019	− 0.026	0.005	2.57 × 10^−06^	0.193	1.000	1	220564510	
Childhood	cg10541930	− 0.010	0.002	4.32 × 10^−08^	0.020	0.020	10	131909085	
	cg25557432	0.014	0.003	1.82 × 10^−07^	0.043	0.086	20	13976117	*MACROD2*
	cg11317171	0.002	0.000	1.34 × 10^−06^	0.190	0.633	19	5623025	*SAFB2;SAFB*
	cg06447795	0.090	0.019	1.62 × 10^−06^	0.190	0.761	20	36793996	*TGM2*
	cg02389501	0.078	0.016	2.59 × 10^−06^	0.191	1.000	7	93757948	
	cg13647052	0.018	0.004	2.82 × 10^−06^	0.191	1.000	12	2800382	*CACNA1C*
Adolescence	cg13974632	0.053	0.010	5.55 × 10^−08^	0.026	0.026	11	27740813	*BDNF*
	cg15810326	0.014	0.003	1.19 × 10^−07^	0.028	0.056	4	148605127	*PRMT10*
	cg16983916	− 0.056	0.011	1.79 × 10^−07^	0.028	0.084	7	156159713	
	cg11510269	0.007	0.001	5.18 × 10^−07^	0.050	0.244	12	123011761	*RSRC2;KNTC1*
	cg17130518	0.052	0.010	5.33 × 10^−07^	0.050	0.251	7	76825482	*CCDC146;FGL2*
	cg06222062	0.095	0.019	6.79 × 10^−07^	0.053	0.319	1	20396690	*PLA2G5*
Lifetime	cg20162381	0.004	0.001	9.22 × 10^−07^	0.159	0.434	12	3310097	*TSPAN9*
	cg20156774	− 0.028	0.006	1.52 × 10^−06^	0.159	0.717	6	32803500	*TAP2*
	cg12888660	0.003	0.001	1.72 × 10^−06^	0.159	0.811	12	122018449	*KDM2B*
	cg15031661	0.006	0.001	1.91 × 10^−06^	0.159	0.897	1	240256603	*FMN2*
	cg17233506	− 0.031	0.006	2.21 × 10^−06^	0.159	1.000	17	46608293	*HOXB1*
	cg18108087	0.010	0.002	2.32 × 10^−06^	0.159	1.000	1	59762112	*FGGY*

**Fig. 2:**
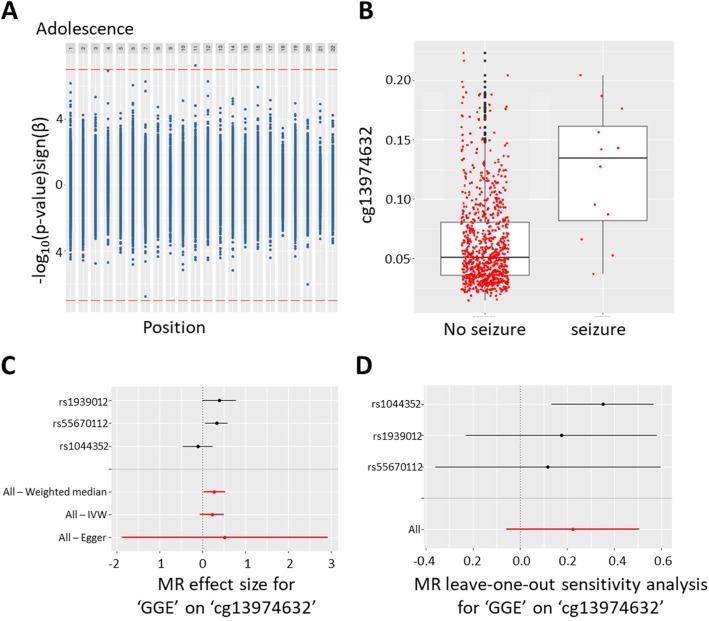
**a** Miami plot displaying the EWAS results by chromosome in adolescence. Positive values on *y*-axis indicate -log(*p* values) of hypermethylated sites, whereas negative values on the *y*-axis indicate -log(*p* values) of hypomethylated sites (the sign of the *y*-axis values has been changed to reflect this). Bonferroni cut-off line in red. **b** Boxplot of methylation levels at the *BDNF*-linked CpG cg13974632 (adjusted for covariates). **c** Causal estimate for the effect of genetic generalized epilepsy on cg13974632 (*BDNF*). Individual SNP results in black and overall causal estimates in red. **d** Leave-one-out analysis of the causal estimate for the effect of genetic generalized epilepsy on cg13974632 (*BDNF*). IVW inverse variance weighted

In adolescence, we found one CpG site that fell below a Bonferroni threshold of 0.05/470,479 = 1.06 × 10^−7^, cg13974632 (beta = 0.053, SE = 0.010, uncorrected *p* value = 5.55 × 10^−8^ and FDR = 0.026), while another two CpG sites passed an FDR threshold only (cg15810326: beta = 0.014, SE = 0.003, uncorrected *p* value = 1.19 × 10^−7^ and FDR = 0.028; cg16983916: beta = − 0.056, SE = 0.011, uncorrected *p* value = 1.79 × 10^−7^ and FDR = 0.028; Fig. 2a). The first CpG site is located in the first exon of the brain-derived neurotrophic factor (BDNF*)*. The experience of seizures was associated with increased DNA methylation at this site (Fig. 2b). The other two CpG sites were located in the first exon of Protein Arginine Methyltransferase 10 (*PRMT10*) and in an intergenic region, respectively.

A sensitivity analysis where we adjusted for fewer cell types to avoid overfitting (i.e., we omitted CD8+ T cell proportions) showed similar association estimates in the probes at FDR < 0.05 in the main EWAS (Additional file 1:Table S2).

No CpG site could be identified to be associated with lifetime seizure experience. Inspection of QQ plots and lambdas close to 1 gave little indication for an inflation of test statistics (Additional file 1: Figure S5).

When testing the association of the significant CpGs at childhood or adolescence across the other age ranges, the effect sizes were much smaller, not significant after correction for multiple comparisons, and for some CpGs in the opposite direction. (Additional file 1: Table S3).

### Replication in the Generation R Study

For replication, we focused on five CpG sites: two that passed FDR correction in childhood and three that passed FDR correction in adolescence. As one of the CpG sites in adolescence was located in the gene *BDNF*, which is a key driver in neuronal growth and has been repeatedly linked to epilepsy [40, 41], we expanded our search space to include all CpG sites annotated to *BDNF* (*n* = 73).

**Fig. 3:**
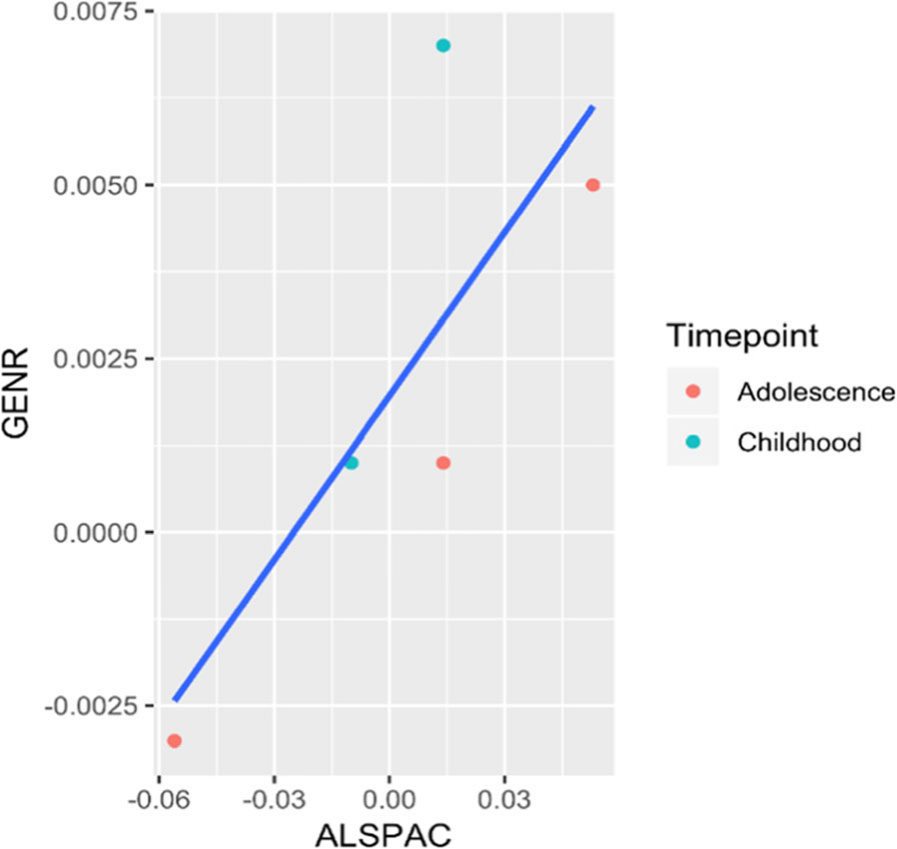
Regression betas in the discovery cohort ALSPAC (*x*-axis) versus betas in the replication cohort Generation R (*y*-axis), plotted for the five most significant probes at each timepoint. The analysis in Generation R was conducted using blood DNA methylation data from children at around 6 years of age and their experience of seizures before that age (*N* = 19 with seizures, *N* = 413 without seizures) and was run including the same covariates as in the discovery cohort

None of the five CpG sites were associated with seizures in Generation R during childhood. Although the direction of effect suggested some degree of concordance, all but one beta coefficient were a factor of 10 smaller and *p* values ranged between 0.1 and 0.8. (Additional file 1: Table S4 and Fig. 3). When ALSPAC and Generation R results for these five probes where meta-analyzed together, all CpGs except for cg16983916 showed evidence of methylation differences (*p* value < 0.05/77 = 0.0006, Additional file 1: Table S4).

Investigating all 73 CpG sites annotated to *BDNF*, none replicated based on a correction for 73 tests. We observed only a weak correlation of all 73 regression betas between cohorts (rho = 0.046, *p* value = 0.70 based on adolescence results in ALSPAC and childhood results in Generation R; Additional file 1: Table S4 and Additional file 1: Figure S6). However, five CpG sites were significant at a nominal level. When ALSPAC and Generation R results for these 73 probes where meta-analyzed together, there was evidence for methylation differences in 2 CpGs (*p* < 0.05/77 = 0.0006, Additional file 1: Table S4). These were cg13974632, the top hit from the EWAS, and cg15313332, 20Kb upstream.

### Cross-tissue concordance in DNA methylation

We queried three independent databases to investigate blood-brain concordance in DNA methylation for all five CpG sites that passed FDR correction in ALSPAC. Based on data from more than 122 pre-mortem blood samples and paired post-mortem brain tissue [42], cross-tissue correlation was strongest for BDNF_cg13974632_ (*r* = 0.39) between blood and brain tissue from the entorhinal cortex, followed by tissue from the prefrontal cortex (*r* = 0.27); Additional file 1: Figure S7A-E. Compared to BDNF_cg13974632_, the remaining four CpG sites showed correlations, which were generally weaker for tissue from the prefrontal and entorhinal cortex. Cross-tissue correlations based on a smaller sample of 16 individuals [43] reported different correlation profiles (Additional file 1: Figure S7F-G). In this dataset, the blood-brain correlation was strongest for MACROD2_cg25557432_ in Brodmann Area BA20 (temporal cortex, rho = 0.48) and BA7 (parietal cortex, rho = 0.43), and for cg15810326 in BA20 (rho = 0.31), whereas there was little evidence of a positive correlation for the other sites in either BA10 (prefrontal cortex), BA20 or BA7.

Cross-tissue blood-brain correlations in a third dataset of 12 epilepsy patients [44] were consistent with the larger dataset, although not discriminating between brain regions (BDNF_cg13974632:_ rho = 0.28, *p* = 0.42; PRMT10_cg15810326_: rho = − 0.0, *p* = 0.94; cg16983916: rho = 0.09, *p* = 0.81; cg10541930: rho = 0.32, *p* = 0.36; MACROD2_cg25557432_: rho = 0.45, *p* = 0.19). Although the correlation coefficients were similar to the larger dataset (*N* = 122), in this smaller dataset (*N* = 12), there was only 9% power to detect a correlation as low as 0.2 at alpha = 0.05 and 26% power to detect a correlation of 0.4.

Based on data available via the Genotype-Tissue Expression (GTEx; www.gtexportal.org) project, we investigated tissue-specific gene expression for genes linked to the five FDR-corrected CpG sites. *BDNF* appeared to be expressed in the brain and other tissues with highest expression in the cerebellum, while very low expression was found in blood. *MACROD2* is predominately expressed in lymphocytes; *PRMT10* is mainly expressed in the ovaries (Additional file 1: Figure S8).

### Mendelian randomization analyses

Uni- and bi-directional two-sample MR was performed to investigate the effect of DNA methylation on seizure occurrence and the effect of seizures on DNA methylation. We used genetic associations with febrile and vaccine-related seizures and genetic associations with epilepsy. The latter more generally includes the types of seizures observed in our study (e.g., febrile and non-febrile) [45]. As full genome-wide summary statistics were only available for epilepsy, but not for febrile and vaccine-related seizures, we could not perform the two-sample MR to estimate the causal effects of methylation on seizures. We identified only one *cis*-mQTL, which could be used as instrument for DNA methylation. In detail, the SNP rs10258194 was associated in *cis* with cg16983916 (effect allele = *T*, beta = 0.25, SD = 0.04, *p* = 2.33 × 10^−10^) after excluding other SNPs due to linkage disequilibrium. For the other CpG sites, either *trans*-associations (further than 1 Mb from the CpG site) or no associations were identified. Two-sample MR indicated only weak evidence for causal effects of DNA methylation at cg1698369 (Additional file 1: Table S5) on epilepsy.

For the reverse (i.e., epilepsy/seizure affecting DNA methylation), there were 9 SNPs to be used as instruments for epilepsy from a previous GWAS meta-analysis on all epilepsies, focal epilepsy, and genetic generalized epilepsy, although only up to four SNPs were used in any one analysis due to availability of summary statistics. Six SNPs were identified as instruments for febrile/vaccine-related seizures, although only 5 were used. Additional file 1: Table S6 shows the results of the two-sample MR analysis performed using different methods to investigate the causal effect of epilepsy on DNA methylation at the five CpG sites identified in the EWAS. For cg13974632 (*BDNF*), there was some evidence for a positive association of genetic generalized epilepsy with increased DNA methylation using the weighted median method (Fig. 2c, d). All methods, including MR-Egger, suggested a positive effect of genetic generalized epilepsy on cg13974632, although these analyses were based on only 3 genetic instruments, and confidence intervals were large, particularly for MR-Egger. The leave-one-out analysis indicated that this effect was not driven by a particular genetic variant, providing little evidence for a violation of MR assumptions. This association did not survive a correction for multiple testing and seemed to be specific for genetic generalized epilepsy (i.e., the effect was not reproduced using focal epilepsy or “any epilepsy” as an exposure). There was no evidence for associations with any other CpG sites.

The two-sample MR analysis of the effects of febrile and vaccine-related seizures on methylation did not show enough evidence of a causal association (Additional file 1: Table S7).

To test the effect of seizure-associated methylation on other health outcomes, we scanned for potentially relevant health traits by performing a PheWAS (association of genotype with all available outcomes) and, subsequently, two-sample MR using the only mQTL available, rs10258194. As we used only one instrument, we could not differentiate whether the associations were due to causal effects or horizontal pleiotropy (i.e., the genetic variant has an effect on the health outcome outside of its effect on DNA methylation at the specific CpG). The analysis revealed little evidence for associations between rs10258194 and other health outcomes, both when analyzed in a hypothesis-free screening PheWAS across all available outcomes (Additional file 1: Table S8) and when analyzed in a two-sample MR with specified outcomes (top outcomes from PheWAS and neurodevelopmental outcomes, Table 3).

**Table 3:** Mendelian randomization analysis. Effects of DNA methylation at cg16983916 on non-epilepsy outcomes (PheWAS and candidate outcomes)

Outcome	Reference	*N*	Beta^a^	S.E.	*P* value (uncorrected)
Weight	UK Biobank^b^	336227	0.016	0.009	0.067
Trunk fat-free mass	UK Biobank^b^	331030	0.025	0.006	3.73 × 10^−05^
Standing height	UK Biobank^b^	336474	0.029	0.007	2.90 × 10^−05^
Years of schooling	Okbay et al. [46]	182286	0.008	0.008	0.317
Childhood intelligence	Benyamin et al. [47]	12441	0.013	0.051	0.797
Autism	Smoller et al. [48]	29415	− 0.008	0.113	0.946
Fluid intelligence score	UK Biobank^b^	108818	0.119	0.036	0.001
College or University degree	UK Biobank^b^	334070	0.013	0.004	0.004

^a^Wald ratio

^b^Unpublished summary statistics from the UK Biobank. https://github.com/Nealelab/UK_Biobank_GWAS and http://www.nealelab.is/blog/2017/9/11/details-and-considerations-of-the-uk-biobank-gwas

## Discussion

In this study, we observed associations between blood DNA methylation and the occurrence of seizures in a longitudinal pregnancy cohort study based in the UK. Effects were specific to childhood and adolescence, with little evidence for a relationship at birth or for lifetime exposure to seizures. However, associations did not replicate in an independent study sample based in the Netherlands. The results are summarized in Additional file 1: Figure S2.

This study has a number of strengths. First, in both ALSPAC and Generation R cohorts, the information on seizures was provided by the parents near the time of occurrence, therefore reducing measurement error and the possibility of recall bias. Secondly, repeated blood sampling at different ages in ALSPAC, including birth, allowed age-specific cross-sectional analyses. Thirdly, these studies have collected extensive information from obstetric records and reported socioeconomic factors allowing adjustment for potential confounders, including birth weight as well as maternal smoking during pregnancy and maternal education. Finally, we used a Mendelian randomization approach as an alternative method to control for unmeasured confounding and examine the direction of observed associations.

Our results in the discovery cohort suggest a link between *BDNF*, a neurotrophin that is highly expressed in the brain, and seizures and epilepsy, at a site where blood and brain DNA methylation levels show correspondence. Mendelian randomization analyses suggest a potential causal effect of seizures on DNA methylation in the *BDNF* gene. Although the implications of the association with *BDNF* are interesting, the association was not replicated. Studies conducted in animal models of epilepsy (reviewed in [49]) observed an upregulation of *BDNF* immediately after experimentally induced seizures. A study conducted on hippocampal tissue from 40 adult patients affected by mesial-temporal lobe epilepsy showed increased or decreased *BDNF* expression, compared to healthy individuals, depending on the region investigated and on the presence of psychiatric comorbidities [50]. Similarly, four isoforms of *BDNF* were found to be highly expressed in brain hippocampal tissue from adult epileptic patients compared to healthy controls, although the effect was not explained by changes in DNA methylation measured in the promoters of isoforms IV and VI [41] . It is to be noted that the association reported in the current study was located further upstream within the first intron of isoforms I, II, and III, based on the latest gene characterization [41, 51]. Moreover, a recent family study investigating genome-wide DNA methylation in peripheral blood, based on 15 trios of parents and their offspring, where the child and one parent, but not the other, were affected by generalized genetic epilepsy, found evidence of neurotrophins involvement, particularly *BDNF*, which was both hyper-and hypomethylated [52]. In our study, we observed hypermethylation in the *BDNF* gene (in the promoter or within introns, depending on the isoform), which would suggest decreased expression. This is in apparent contrast with some of the previous studies, but in line with our Mendelian randomization analysis that also showed some evidence of seizure-induced hypermethylation in the *BDNF* gene when using generalized genetic epilepsy as the exposure. The apparently contrasting findings in the BDNF gene and the lack or reproducibility of this association could be explained by the fact that BDNF expression in blood is very low, but follow-up investigations in biological systems with multi-tissue characterization are required to elucidate this further.

We also show potential associations with other methylation sites that have not been previously observed, specifically in the *MACROD2*, the *PRMT10* genes, and two intergenic sites. Single nucleotide polymorphisms within *MACROD2* have previously been associated with autism, although with rather weak evidence [53], and other brain-related traits such as intelligence and mathematical abilities [54]. Individuals with *de novo* mutations in *FBXO11,* an analogous of *PRMT10,* have been reported to show intellectual disability and autism [55]. Although both genes are predominantly expressed in non-neural tissues, these studies suggest that *MACROD2 and PRMT10* could play a role in brain functioning, and their methylation status could plausibly be involved in seizures. However, as these associations were not replicated, the involvement of DNA methylation in blood at these sites needs to be further investigated.

The results of this study have to be seen in light of the following limitations. First, despite this being the largest epigenome-wide association study in childhood/adolescence, the sample size was small considering the low prevalence of seizures in the general population. Second, our initial findings did not replicate in data from an independent cohort. However, this could also indicate that the associations are specific to the time window examined in the discovery sample. For instance, the association with *BDNF* methylation was specific to the adolescence time point, while in Generation R data was limited to childhood. Thirdly, we have relied on DNA methylation measured in blood whereas seizures occur within the brain. Availability of brain tissue for epidemiological research is very limited, and future studies could follow-up our findings in animal models or post-mortem brain tissues. Finally, the Mendelian randomization analyses relied on a small number of mQTLs (3 for epilepsy to *BDNF* methylation and 1 for methylation to epilepsy and other health outcomes) and therefore did not allow for sensitivity analyses aimed at ruling out bias due to horizontal pleiotropy.

## Conclusions

In conclusion, we show that the evidence of a link between seizures and blood DNA methylation in childhood and adolescence is weak. Our study highlights the challenges of conducting epigenome-wide association studies of seizures across different developmental periods and warrants a careful analysis of the data in view of the limitations of such study.

## Supplementary information

**Additional file 1:** **Figure S1.** Overlap of cases across time points. **Figure S2.** Overview of results. All models were adjusted for covariates as specified in the main text (including cell composition and surrogate variables). In MR analyses, epilepsy was chosen as a trait due to the availability of GWAS summary data as well as febrile and MMR-vaccine-related seizures. **Figure S3.** Plots displaying bivariate correlations between all variables included in the final models: (A) at birth, (B) during childhood and (C-D) adolescence (cross-sectional and lifetime seizure exposure, respectively). **Figure S4.** Miami plots displaying EWAS results: (A) at birth, (B) during childhood and (C-D) adolescence (cross-sectional and lifetime seizure exposure, respectively). The sign of the y-axis (-log(P-values) has been changed to indicate positive and negative changes in DNA methylation. Bonferroni cut-off line in red. **Figure S5.** Quantile-quantile plots displaying potential test statistic inflation in the final models (A) at birth, (B) during childhood and (C-D) adolescence (cross-sectional and lifetime seizure exposure, respectively). Lambda was calculated using the regression method. **Figure S6.** Differences in DNA methylation according to experienced seizures in ALSPAC and Generation R (top panel) in the BDNF gene. The bottom panel shows the location of the transcripts reverse strand. **Figure S7.** Cross-tissue correspondence of CpG sites, passing FDR correction in ALSPAC, based on data available at A-E) https://epigenetics.essex.ac.uk/bloodbrain and F-G) https://redgar598.shinyapps.io/BECon. PFC = prefrontal cortex; STG = superior temporal gyrus; EC = entorhinal cortex; CER = cerebellum. **Figure S8.** Tissue-specific expression of BDNF, MACROD2 and PRMT10, based on data available at www.gtexportal.org. **Table S1.** Sample descriptives of Generation R. **Table S2.** Association estimates for FDR<0.05 probes re-analyzed with adjustment for 5 cell types. **Table S3.** Association of DNA methylation and the occurrence of seizures at CpGs with FDR<0.05 across all models. **Table S4.** Replication in Generation R and meta-analysis. **Table S5.** Two-sample MR analysis of the effect of DNA methylation on epilepsy (method=Wald ratio). **Table S6.** Two-sample MR analysis of the effect of epilepsy on DNA methylation. **Table S7.** Two-sample MR analysis of the effect of febrile and vaccine-related seizures on DNA methylation. **Table S8.** Top 50 associations with rs10258194 in MR_Base.

## Data Availability

The participants’ data used in this study are not publicly available due to privacy restrictions. However, the data may be available upon request following the relevant procedures for ALSPAC (http://www.bristol.ac.uk/alspac/researchers/access/) and Generation R (https://generationr.nl/researchers/collaboration/).

## Abbreviations

ALSPAC: Avon longitudinal study of parents and children
ARIES: Accessible resource for integrative epigenomic studies
BDNF: Brain-derived neurotrophic factor
CpG: Cytosine-guanine dinucleotide
EWAS: Epigenome-wide association study
FDR: False discovery rate
GWAS: Genome-wide association study
IQR: Interquartile range
MMR: Mumps measles rubella
mQTL: Methylation quantitative trait loci
MR: Mendelian randomization
PheWAS: Phenome-wide association study
SM: Supplementary material
SNP: Single-nucleotide polymorphism
SV: Surrogate variable
