# Supplementary material for: Epigenome-wide association study of seizures in childhood and adolescence
Source: Clin Epigenetics. 2020 Jan 8;12:8. doi: 10.1186/s13148-019-0793-z (PMC6950851; doi:10.1186/s13148-019-0793-z)
Supplement: Supplementary file 1 — Additional file 1: Figure S1. Overlap of cases across time points. Figure S2. Overview of results. All models were adjusted for covariates as specified in the main text (including cell composition and surrogate variables). In MR analyses, epilepsy was chosen as a trait due to the availability of GWAS summary data as well as febrile and MMR-vaccine-related seizures. Figure S3. Plots displaying bivariate correlations between all variables included in the final models: (A) at birth, (B) during childhood and (C-D) adolescence (cross-sectional and lifetime seizure exposure, respectively). Figure S4. Miami plots displaying EWAS results: (A) at birth, (B) during childhood and (C-D) adolescence (cross-sectional and lifetime seizure exposure, respectively). The sign of the y-axis (-log(P-values) has been changed to indicate positive and negative changes in DNA methylation. Bonferroni cut-off line in red. Figure S5. Quantile-quantile plots displaying potential test statistic inflation in the final models (A) at birth, (B) during childhood and (C-D) adolescence (cross-sectional and lifetime seizure exposure, respectively). Lambda was calculated using the regression method. Figure S6. Differences in DNA methylation according to experienced seizures in ALSPAC and Generation R (top panel) in the BDNF gene. The bottom panel shows the location of the transcripts reverse strand. Figure S7. Cross-tissue correspondence of CpG sites, passing FDR correction in ALSPAC, based on data available at A-E) https://epigenetics.essex.ac.uk/bloodbrain and F-G) https://redgar598.shinyapps.io/BECon. PFC = prefrontal cortex; STG = superior temporal gyrus; EC = entorhinal cortex; CER = cerebellum. Figure S8. Tissue-specific expression of BDNF, MACROD2 and PRMT10, based on data available at www.gtexportal.org. Table S1. Sample descriptives of Generation R. Table S2. Association estimates for FDR<0.05 probes re-analyzed with adjustment for 5 cell types. Table S3. Association of DNA methylation and the [file 13148_2019_793_MOESM1_ESM.docx]

**Epigenome-wide association study of seizures in childhood and adolescence**

**Supplemental info**

Doretta Caramaschi, PhD^1,2^ *, Charlie Hatcher, MSc^1,2^, Rosa H. Mulder, MSc^3,4,5^, Janine F. Felix, PhD^5,6^, Charlotte A. M. Cecil, PhD^4,6^, Caroline L. Relton, PhD^1,2^, Esther Walton, PhD^1,2,8^.

^1^ Bristol Medical School, Population Health Sciences, University of Bristol, UK

^2^ Medical Research Council Integrative Epidemiology Unit, University of Bristol, UK

^3^ Institute of Education and Child Studies, Leiden University, The Netherlands

^4^ Department of Child and Adolescent Psychiatry/Psychology, Erasmus MC, University Medical Center Rotterdam, Rotterdam, The Netherlands

^5^ Generation R Study Group, Erasmus MC, University Medical Center Rotterdam, Rotterdam, The Netherlands

^6^ Department of Pediatrics, Erasmus MC, University Medical Center Rotterdam, Rotterdam, the Netherlands

^7^ Department of Epidemiology, Erasmus MC, University Medical Center Rotterdam, Rotterdam, The Netherlands

^8^ Department of Psychology, University of Bath, UK

**Figure S1**. Overlap of cases across time points.

**Figure S2**. Overview of results. Epilepsy was chosen as a trait (rather than seizures) due to the availability of GWAS summary data on epilepsy based on UK Biobank, International League Against Epilepsy Consortium on Complex Epilepsies as well as febrile and MMR-vaccine-related seizures.

Covariates used in each model (SV=surrogate variable):

M1: age + sex + birthweight + gestational age + maternal smoking + maternal education + nucleated red blood cells + granulocytes + monocytes + natural killer cells + B cells + CD4(+)T cells + CD8(+)T cells + SV1 + … + SV15

M2: age + sex + maternal smoking + maternal education + granulocytes + monocytes + natural killer cells + B cells + CD4(+)T cells + CD8(+)T cells + SV1 + … + SV13

M3: age + sex + maternal smoking + maternal education + granulocytes + monocytes + natural killer cells + B cells + CD4(+)T cells + CD8(+)T cells + SV1 + … + SV14

M4: age + sex + maternal smoking + maternal education + granulocytes + monocytes + natural killer cells + B cells + CD4(+)T cells + CD8(+)T cells + SV1 + … + SV14

M2 (Generation R): age + sex + maternal smoking + maternal education + granulocytes + monocytes + natural killer cells + B cells + CD4(+)T cells + CD8(+)T cells + SV1 + … + SV13

**Figure S3**. Plots displaying bivariate correlations between all variables included in the final models: (A) at birth, (B) during childhood and (C-D) adolescence (cross-sectional and lifetime seizure exposure, respectively).

**Figure S4**. Miami plots displaying EWAS results: (A) at birth, (B) during childhood and (C-D) adolescence (cross-sectional and lifetime seizure exposure, respectively).Positive values on y-axis indicate -log(P-values) of hypermethylated sites, whereas negative values on the y-axis indicate -log(P-values) of hypomethylated sites (the sign of the y-axis values have been changed to reflect this). Bonferroni cut-off line in red.

**Figure S5**. Quantile-quantile plots displaying potential test statistic inflation in the final models (A) at birth, (B) during childhood and (C-D) adolescence (cross-sectional and lifetime seizure exposure, respectively). Lambda was calculated using the regression method.


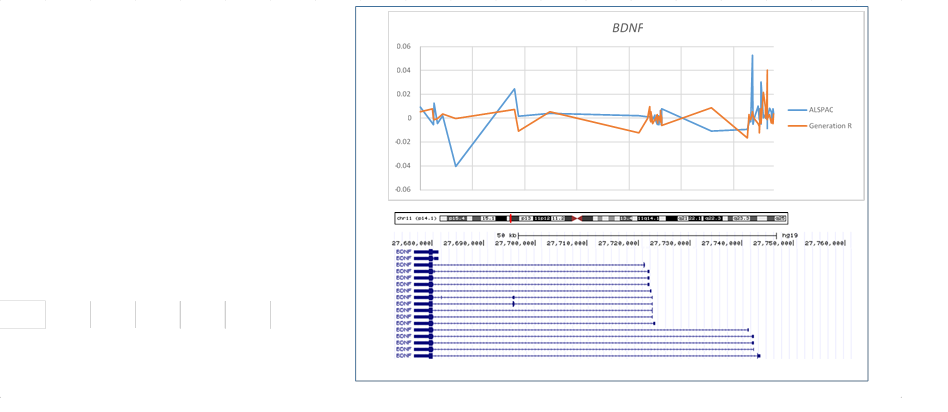


**Figure S6**. Differences in DNA methylation according to experienced seizures in ALSPAC and Generation R (top panel) in the *BDNF* gene. The bottom panel shows the location of the transcripts reverse strand.

**Figure S7**. Cross-tissue correspondence of CpG sites, passing FDR correction in ALSPAC, based on data available at A-E) https://epigenetics.essex.ac.uk/bloodbrain and F-G) https://redgar598.shinyapps.io/BECon. PFC = prefrontal cortex; STG = superior temporal gyrus; EC = entorhinal cortex; CER = cerebellum.

**Figure S8**. Tissue-specific expression of *BDNF*, *MACROD2* and *PRMT10*, based on data available at www.gtexportal.org.

**Table S1**. Sample descriptives of Generation R.

**Table S2**. Association estimates for FDR<0.05 probes re-analyzed with adjustment for 5 cell types.

|  | **probeID** | **BETA** | **SE** | **P-value** | **FDR** | **Bonferroni** | **Gene** |
| --- | --- | --- | --- | --- | --- | --- | --- |
| Childhood | cg10541930 | -0.010 | 0.002 | 4.82E-08 | 0.023 | 0.023 |  |
|  | cg25557432 | 0.014 | 0.003 | 2.06E-07 | 0.048 | 0.097 | *MACROD2* |
| Adolescence | cg13974632 | 0.051 | 0.0096 | 1.24E-07 | 0.032 | 0.058 | *BDNF* |
|  | cg16983916 | -0.056 | 0.0106 | 1.88E-07 | 0.032 | 0.088 | *PRMT10* |
|  | cg15810326 | 0.014 | 0.0026 | 2.05E-07 | 0.032 | 0.096 |  |

**Table S3.** Association of DNA methylation and the occurrence of seizures at CpGs with FDR<0.05 across all models.

**Table S4**. Replication in Generation R and meta-analysis.

**Table S5**. Two-sample MR analysis of the effect of DNA methylation on epilepsy (method=Wald ratio).

**Table S6**. Two-sample MR analysis of the effect of epilepsy on DNA methylation.

GGE= Genetic generalized epilepsy

**Table S7**. Two-sample MR analysis of the effect of febrile and vaccine-related seizures on DNA methylation.

**Table S8**. Top 50 associations with rs10258194 in MR_Base.
